# Supplementary figures and images for: Ectopic Pregnancy and T-Cell Lymphoma in a Eurasian Red Squirrel (Sciurus vulgaris): Possible Comorbidity and a Comparative Pathology Perspective
Source: Animals (Basel). 2024 Feb 27;14(5):731. doi: 10.3390/ani14050731 (PMC10930950; doi:10.3390/ani14050731)

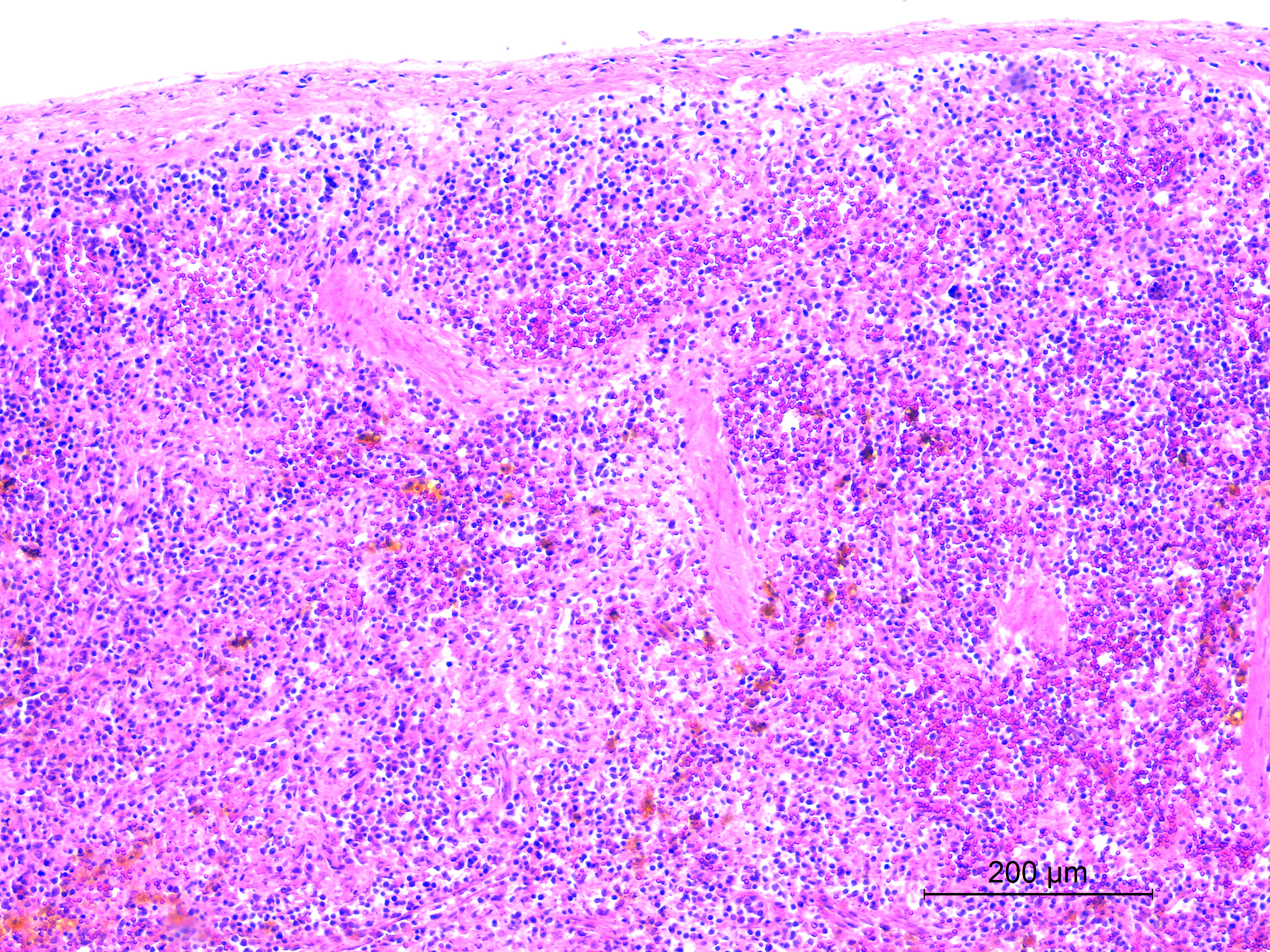

Supplement: Supplementary file 1 [file animals-14-00731-s001.zip › Figure S1.tif]

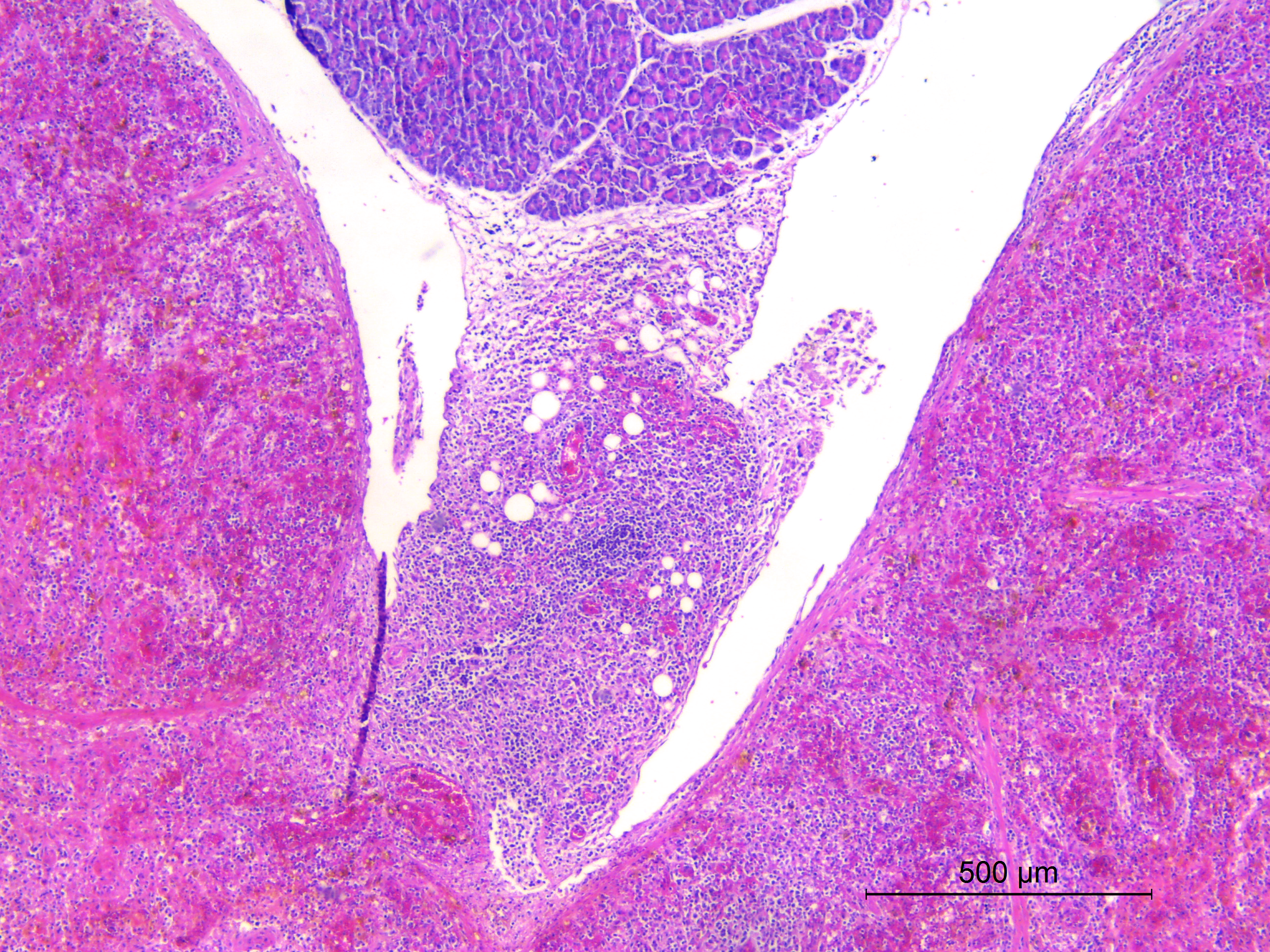

Supplement: Supplementary file 1 [file animals-14-00731-s001.zip › Figure S2.tif]

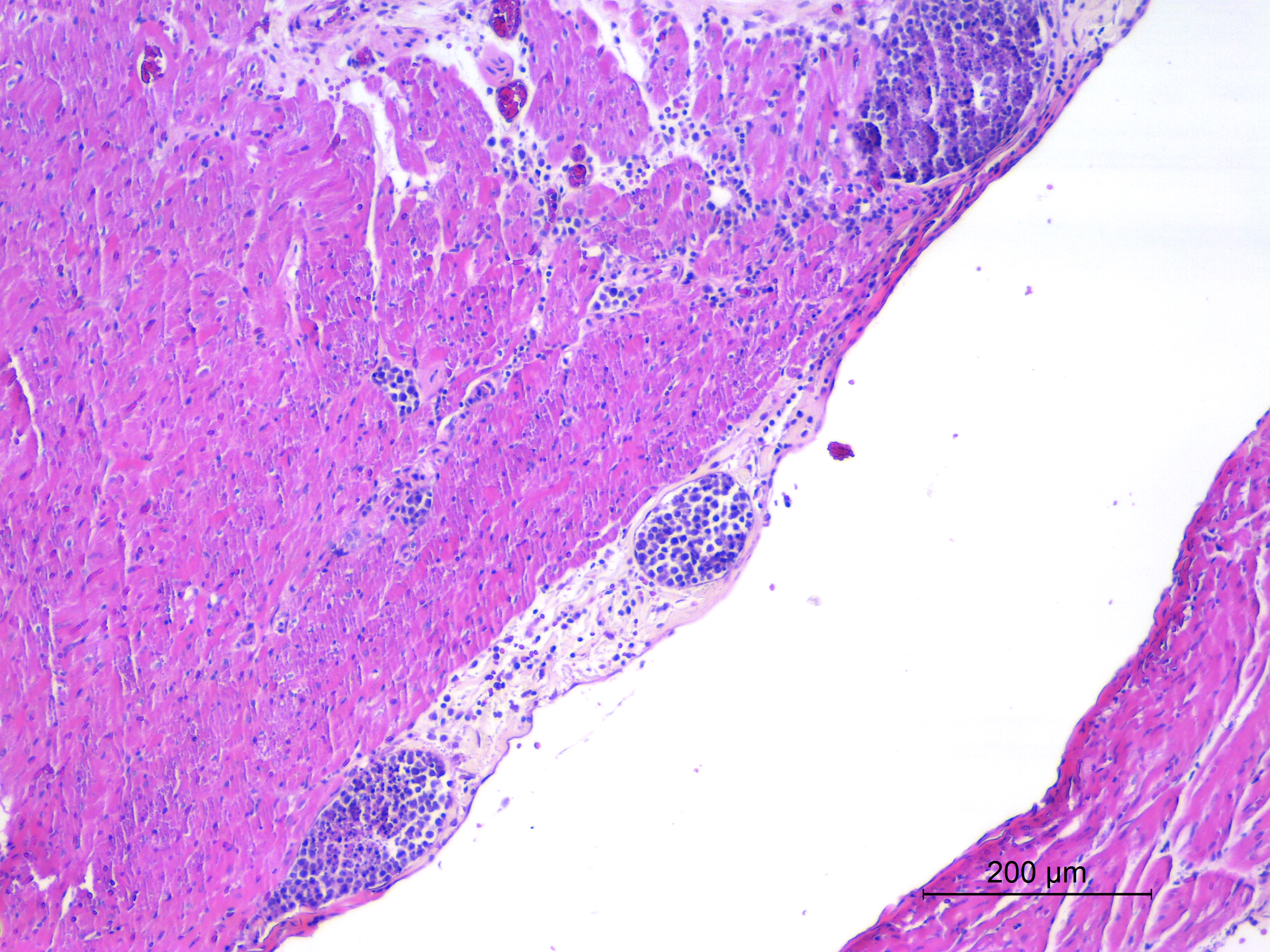

Supplement: Supplementary file 1 [file animals-14-00731-s001.zip › Figure S3.tif]
